# Supplementary material for: Step-to-step variability indicates disruption to balance control when linking the arms and legs during treadmill walking
Source: PLoS One. 2022 Mar 23;17(3):e0265750. doi: 10.1371/journal.pone.0265750 (PMC8942237; doi:10.1371/journal.pone.0265750)
Supplement: S2 File — This file contains the protocol and graphs of the sensitivity analyses performed in GPower. (DOCX) [file pone.0265750.s002.docx]

**Vega, Huang, and Arellano (2022)**

**Sensitivity analyses**

**Sensitivity analysis 1 GPower protocol:**

**t tests -** Means: Difference between two dependent means (matched pairs)

**Analysis:** Sensitivity: Compute required effect size

**Input:** Tail(s) = One

α err prob = 0.05

Power (1-β err prob) = 0.80

Total sample size = 8

**Output:** Noncentrality parameter δ = 2.7666432

Critical t = 1.8945786

Df = 7

**Effect size dz = 0.9781561**


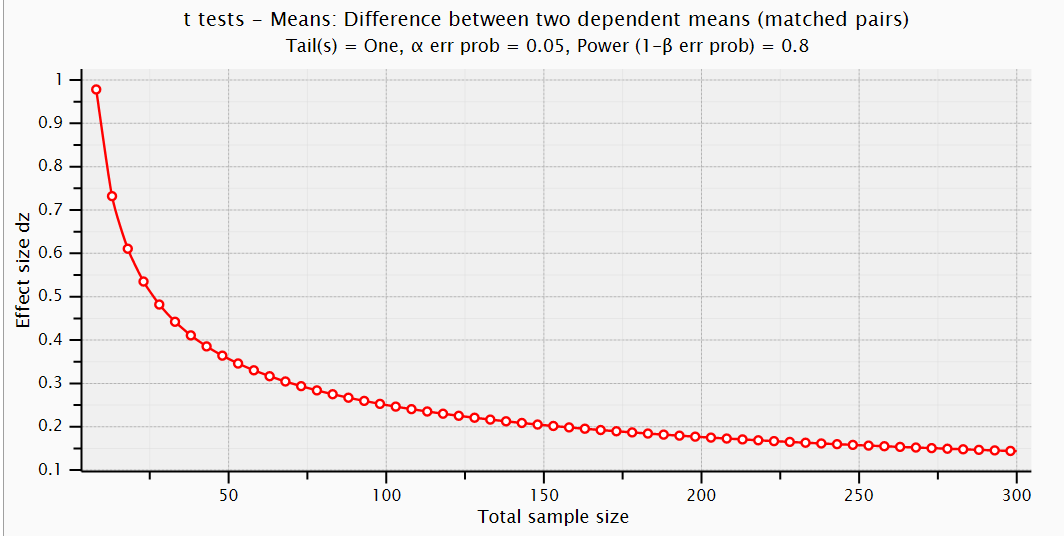


**Figure 1.** Sensitivity analysis for determining the effect size that would be detectable across various sample sizes with 80% power, α = 0.05, and a one-tailed dependent t-test. In our study of n = 8, the detectable effect size for our parametric variables step width and step time variability would yield a detectable effect size of at least 0.98 or above.

**Sensitivity analysis 2 GPower protocol:**

**t tests -** Means: Wilcoxon signed-rank test (matched pairs)

**Options:** A.R.E. method

**Analysis:** Sensitivity: Compute required effect size

**Input:** Tail(s) = One

Parent distribution = Normal

α err prob = 0.05

Power (1-β err prob) = 0.80

Total sample size = 8

**Output:** Noncentrality parameter δ = 2.7844074

Critical t = 1.9101730

Df = 6.6394373

**Effect size dz = 1.0074003**


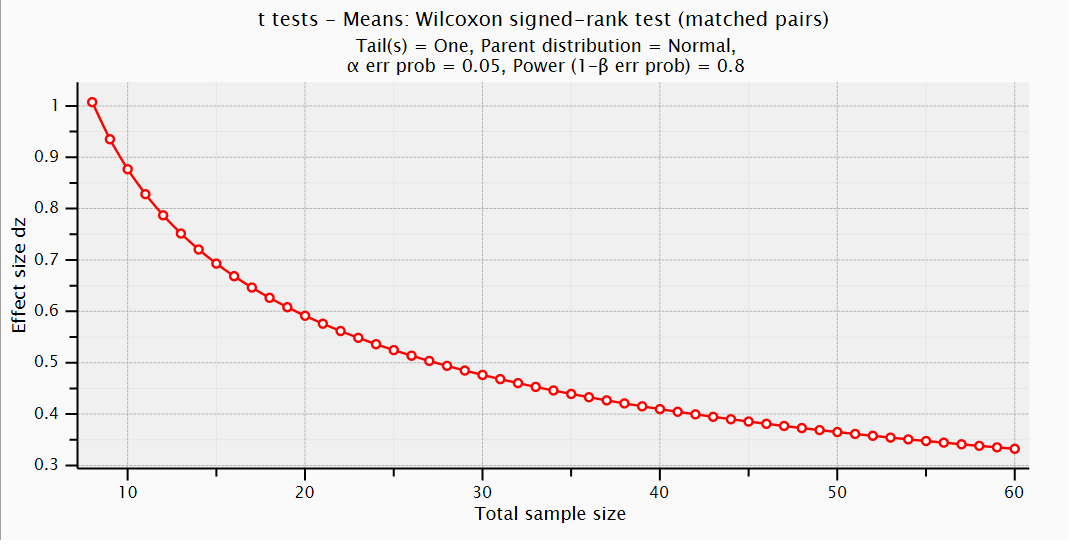


**Figure 2.** Sensitivity analysis for determining the effect size that would be detectable across various sample sizes with 80% power, α = 0.05, and a one-tailed Wilcoxon signed-rank test (matched pairs). In our study of n = 8, the detectable effect size for our non-parametric variable step length variability would yield a detectable effect size of at least 1.00 or above.

**Sensitivity analysis 3 GPower:**

**
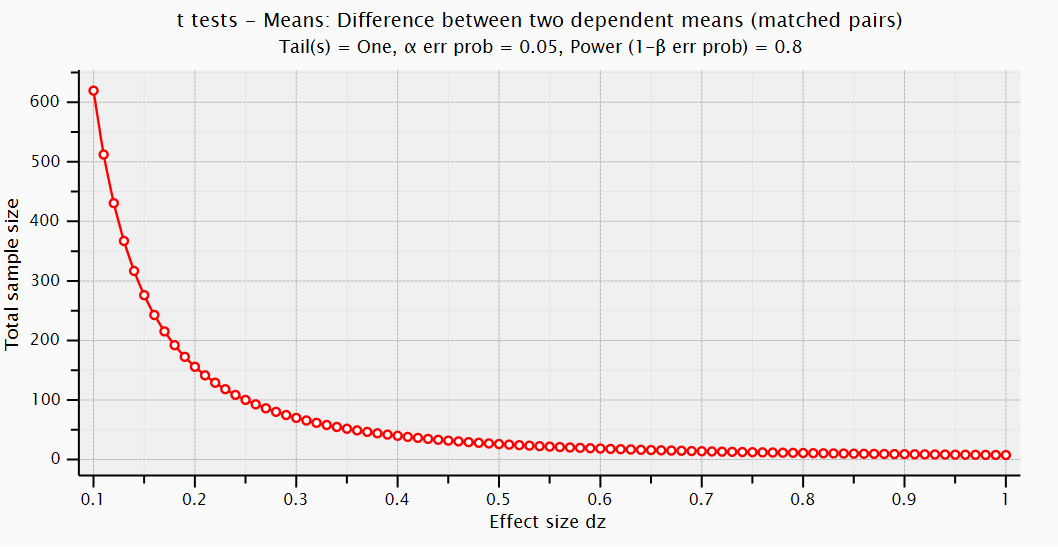
**

**Figure 3.** Sensitivity analysis for determining the sample size needed across various effect sizes with 80% power, α = 0.05, and a one-tailed dependent t-test. Based on the effect sizes gathered from the literature for step time, length and width variability (ES = 0.486, 0.369 and 0.845, respectively. Please see S1 File for reference), the analysis suggests a study would require between 10 and 49 subjects to be able detect these effect sizes with sufficient power.
