# Supplementary material for: Step-to-step variability indicates disruption to balance control when linking the arms and legs during treadmill walking
Source: PLoS One. 2022 Mar 23;17(3):e0265750. doi: 10.1371/journal.pone.0265750 (PMC8942237; doi:10.1371/journal.pone.0265750)
Supplement: Attachment — Submitted filename: Response to Reviewers.docx [file pone.0265750.s003.docx]

**Response to Editor and Reviewer’s Comments**

**PONE-D-21-31057**

**Step-to-step variability indicates disruption to balance control when linking the arms and legs during treadmill walking [Revised Title]**

**PLOS ONE**

**Journal requirements**

When submitting your revision, we need you to address these additional requirements.

**1**. Please ensure that your manuscript meets PLOS ONE's style requirements, including those for file naming. The PLOS ONE style templates can be found at

[https://journals.plos.org/plosone/s/file?id=wjVg/PLOSOne_formatting_sample_main_body.pdf](https://urldefense.com/v3/__https:/journals.plos.org/plosone/s/file?id=wjVg*PLOSOne_formatting_sample_main_body.pdf__;Lw!!LkSTlj0I!QwpvSkyUpo9JsNLi-Bcnzk6duDckwEE0UJqciDRzeWDdYaYs_aT9ndRoay3OMo4$) and

[https://journals.plos.org/plosone/s/file?id=ba62/PLOSOne_formatting_sample_title_authors_affiliations.pdf](https://urldefense.com/v3/__https:/journals.plos.org/plosone/s/file?id=ba62*PLOSOne_formatting_sample_title_authors_affiliations.pdf__;Lw!!LkSTlj0I!QwpvSkyUpo9JsNLi-Bcnzk6duDckwEE0UJqciDRzeWDdYaYs_aT9ndRoGsR9FyI$)

**Response: We have updated our manuscript to meet PLOS ONE’s style requirements.**

**2.** Please amend your current ethics statement to address the following concerns:

a) Did participants provide their written or verbal informed consent to participate in this study?

b) If consent was verbal, please explain i) why written consent was not obtained, ii) how you documented participant consent, and iii) whether the ethics committees/IRB approved this consent procedure.

**Response: We have amended our ethics statement with following statement:**

**A health screen form was completed, reviewed, and signed by the subject the day of the experimental session. In addition, they read and signed an informed consent document. All subjects provided written informed consent. This study was reviewed and approved by the University of Houston Institutional Review Board.**

**3.** We note that you have stated that you will provide repository information for your data at acceptance. Should your manuscript be accepted for publication, we will hold it until you provide the relevant accession numbers or DOIs necessary to access your data. If you wish to make changes to your Data Availability statement, please describe these changes in your cover letter and we will update your Data Availability statement to reflect the information you provide.

**Response: We have made the data available in an excel sheet, see S1 File.**

**Review Comments to the Author**

Please use the space provided to explain your answers to the questions above. You may also include additional comments for the author, including concerns about dual publication, research ethics, or publication ethics. (Please upload your review as an attachment if it exceeds 20,000 characters)

***Reviewer #1*:**

In this paper the authors conduct a secondary analysis on a device they previously developed that may be useful as a gait rehabilitation device in the future. Specifically, they previously saw metabolic benefits to mechanically coupling the arms and the legs during treadmill walking, but this new device also lead to abnormal arm swing. Thus, in the present study the authors sought to determine if there were changes in the stepping pattern while mechanically coupling the arms and legs since significant disruptions to normal walking could limit the device’s usefulness in a patient population.

Overall, I am satisfied with the manuscript as it was initially submitted, and I have no major suggestions for improvement. My recommendation is to accept the paper. The authors frame the scope of this paper well. The methods are sound, and the conclusions they draw are reasonable. I look forward to seeing the future work. My three comments below are minor and do not need to be addressed in the paper itself. They are more curiosities than critiques.

**Response: Thank you for your careful review and supportive feedback.**

**Comment 1:** I’m curious what the effect size was for step length variability. The p-value was not quite significant, but Figure 3 shows a decent effect in all but one subject, as you mentioned in the caption. In the ideal world, your device would provide metabolic benefits without any changes to the stepping pattern. Thus, for the purposes of this paper (identifying potential disruptions to normal walking), I think it is reasonable to consider a p-value of 0.062 as a potential effect. Step length and step length variability are the variables I would a priori assume to be most affected (as opposed to step width) since the rope-pulley system directly pulls on the feet in the anteroposterior direction as opposed to mediolaterally.

**Response: We appreciate your curiosity and agree that the effect size may be more telling than reporting the p-value in itself. While we initially reported effect sizes only if the p -value was significant, we have revised the results section to include effect sizes for all variables examined. The effect size for step length variability is 0.385 as calculated using the Rosenthal effect size since this variable did not meet the assumption of normality. We agree that one could consider a p-value of 0.062 as a potential effect. Based on its effect size and how it compares to the literature, we conclude that there may have been a minimal to moderate disruption to walking balance in the anterior-posterior direction. We have added this interpretation to the Discussion.**

**During this process, we realized that we made a mistake with our previously reported effect sizes. We were reporting the “standardizer” from our SPSS output and not the “point-estimate” which is the actual effect size. Specifically, the effect size for step time variability was a lot greater than initially reported. We apologize for this mistake. We have now corrected and revised our interpretations accordingly. We have noted in the Discussion that our small sample size is a limitation and should be considered when interpretating our findings.**

**Comment 2:** In future work, I would be interested in exploring connecting the arms to the contralateral legs. You referenced previous work by Zehr who has explored the neural coupling between the arms and legs. I am only somewhat aware of his work, not the major findings nor how they directly relate to your current work. I would be interested to know from a neuro-rehab perspective if linking arms and legs ipsilaterally versus contralaterally is expected to be more beneficial, for example for individuals that have experienced a stroke. It would be slightly more difficult to implement than your current setup, but it should still be reasonably possible to change the mechanical coupling to be contralateral if there were a specific benefit to connecting them that way.

**Response: Yes, this is an interesting point. We have also thought about other potential designs where we could link the arms and legs contralaterally, as this might promote neural coupling in different ways. Thank you for sharing with us your curiosity and insight.**

**Comment 3:** In future work I might also suggest adding an elastic band or other spring mechanism between the ropes and the contact points on the body. That would allow you to dampen or limit the forces between the arms and legs which may help people more quickly learn how to walk in the device.

**Response: Thank you for the insight and suggestion. That is definitely a design feature that we will be exploring in the future. We had thought about adding a springy mechanism to possibly induce a power amplification effect, which could lead to greater reductions in propulsive forces and increase the metabolic benefits. However, we never thought about using it to dampen the forces as a way to help with learning. That is a very interesting way to think about a springy mechanism. It will be interesting to see how this might work in our future experiments.**

***Reviewer #2:***

**1)** Briefly describe the rationale and mechanism of the rope-pulley system. It seems to be beneficial for reducing the mechanical cost of walking based on a previous study, but there is no description of why it is important and by what mechanism it helps human gait. Readers should understand this from the current paper without visiting the previous study. Briefly describe this in the introduction.

**Response: Thank you for the helpful suggestion. We have added to the Introduction that the rope-pulley system is mechanically beneficial (propels the body forward) and the mechanism for how it helps human walking (active use of the arms assists with generating propulsive forces). Please refer to 1^st^ paragraph in the clean version of the manuscript. To avoid reiterating excerpts from our previous paper, we kept this description as concise as possible. And for the interested reader, we have referenced our previous paper for more in-depth mechanism of how the rope-pulley system worked.**

**2)** Other questions regarding the rope-pulley system: Why is reducing net metabolic power important when walking? Does the system increase arm swing range of motion or velocity?

**Response: Thank you again for pointing out another important aspect to include in our paper. We have now addressed the importance of reducing net metabolic power during walking in the Introduction. In regard to your second question, the system increased arm swing range. Specifically, it increased shoulder protraction and increased elbow joint flexion/extension. We did not analyze arm swing velocity as this manuscript was focused on understanding changes in step timing and kinematics. However, analyzing arm swing velocity is an interesting thought and we will consider that type of analyses in our future experiments.**

**3)** I have a concern regarding the statistical power of this study. Although the difference in stride length variability was insignificant, it was close to be significant, and all but one participant showed increased stride length variability (Figure 3). I suspect a low statistical power of this result along with low effect size in other results and thus suggest increasing the sample size or justify the sample size (n = 8) by conducting a power analysis.

**Response: Thank you for raising your concerns. We reported the effect size for step length variability (Rosenthal ES = 0.385) and when compared to the literature, we conclude that there may have been minimal to moderate disruption in the anterior-posterior direction. We agree with your suspicion that the insignificant result may be due to the small sample size. Since this paper is a secondary analysis from our original study that focused on metabolic power and mechanics, we did not conduct a priori power analysis. With that in mind, we make it clear that this is secondary analysis in the Introduction. For transparency, we also state that the small sample size is a limitation and that in a future study, we will recruit a larger sample size.**

**4)** The summary of findings based on the low sample size also raises concerns. The authors repeatedly states that there was no increase in either step width variability or step length variability which indicate no spatial components of walking balance. This conclusion is a big leap and could be biased without warranting the statistical power.

**Response: Thank you again for raising these concerns. While we initially reported effect sizes only if the p-value was significant, we have revised the results section to include the effect sizes step width variability and step length variability which are 0.146 and 0.385, respectively. In the discussion, we compare the effect sizes to what we could find in the literature with adequate sample sizes and statistically significant results and conclude minimal to moderate disruption in the spatial component of walking balance.**

**5)** The authors’ interpretation of small effect size also raises concerns. The authors interpret the small effect size for step time variability to indicate a minimal disruption to the temporal component of walking balance. However, the small effect size could be due to the small sample size not because of the minimal disruption. Verify that the degree of differences in step time variability is minimal before reaching to a conclusion.

**Response: This is an excellent point. We agree that it is possible that the small effect size is due to a small sample size and not due to minimal disruption. This motivated us to revisit our effect size and in doing so, we found a mistake. We were reporting the “standardizer” from our SPSS output and not the “point-estimate” which is the actual effect size. Therefore, the effect size for step time variability was a lot greater than initially reported. We apologize for this mistake. We have now corrected this mistake and revised our interpretations accordingly. To come to a reasonable interpretation as to the magnitude of the effect size, we revisited key papers available in the literature that had an adequate sample size and detected significant differences in these stepping parameters. Overall, we conclude that a moderate disruption to the temporal aspect of walking balance occurred. This is contrary to our initial conclusion of “minimal disruption” which was based on our wrongly reported “effect size”. We agree that our small sample size is still of concern and should be addressed in future studies by using a larger sample size. To remedy this limitation, we do our best by comparing our effect sizes to the limited data that is available in the clinical and gait rehabilitation literature. We thank you for the helpful feedback and hope our revisions and updated interpretations have satisfied your key concerns.**
