# Supplementary material for: Step-to-step variability indicates disruption to balance control when linking the arms and legs during treadmill walking
Source: PLoS One. 2022 Mar 23;17(3):e0265750. doi: 10.1371/journal.pone.0265750 (PMC8942237; doi:10.1371/journal.pone.0265750)
Supplement: Attachment — Submitted filename: Response to Reviewers FINAL.docx [file pone.0265750.s004.docx]

**Response to Reviewer’s Comments**

**Reviewer #1:**

I am satisfied with the current state of the paper and recommend it is accepted for publication.

**Response: Thank you so much for taking the time to review our manuscript. Your previous comments contributed to the improvement of our manuscript.**

**Comment 1:** I commend the authors for catching the mistake with reporting the effect size estimate.

**Response: Thank you. We appreciate the leniency and acknowledgement, and rest assured that we have learned from this mistake!**

**Comment 2:** In future studies it might be worthwhile to add a quick subjective measure of participants' self-perceived walking balance. That will help disambiguate meaningful disruptions to balance from mild or imperceptible disruptions.

**Response: Yes, we absolutely agree. In addition to measuring subject’s self-perceived walking balance, it would also be beneficial to capture other aspects of their subjective experience. We think it would be interesting to also measure their confidence and/or fear of falling. It would also be interesting to measure their perceived effort of the arms and legs (and compare it to their EMG data), as well as their perceived overall effort (and compare it to their whole-body energetics). These are all interesting aspects to consider in future studies. Thanks again for sharing your thoughts.**

**Reviewer #2:**

**Comment 1:** The authors conducted a secondary data analysis from a parent study to further examine whether the rope pulley system disrupts walking balance in healthy adults. They hypothesized that the rope pulley system may increase the variability of at least one of step width, step length, and step time which may indicate the disruption of walking balance. The result indicates increased variability in step time (M = 5 ms, p < 0.05, Cohen’s d = 1.286) but not in other parameters. The authors conclude that healthy subjects experienced minimal to moderate disruptions in walking balance when using with this device. This study addresses an interesting topic, and the manuscript is well written. However, given that the analysis is based on a small sample size and the significant result was found in only one of three variables of interest, there remains a couple of shortcomings in this study to be published under the current condition.

**Response: Thank you for taking the time to review our manuscript and we appreciate your interest and comments. As you stated, this is a secondary analysis from our previous data set. We previously investigated the energetics and mechanics of using the arm-leg rope-pulley system during treadmill walking in healthy subjects and at that time, we did not consider balance. It was only after we visualized the motion capture data that we started to think about balance to provide a more comprehensive understanding of mechanically coupling the arms and legs during walking. Unfortunately, at completion of our original study, we closed the protocol with our IRB and therefore, we were limited to using the previous existing data set since recruiting additional subjects was not possible. With this in mind, we have treated the small sample size with a critical lens by using the appropriate statistical approaches and interpreting our effect sizes to those values reported in the literature, which we have found to be limited. We also believe the present study helps inform future steps of the best approach to mechanically couple the arms and legs during walking, all while keeping balance demands and the target clinical population (e.g., spinal cord injury) in mind.**

**In the manuscript, we state that this is a secondary analysis:**

**Lines 72-74: “Therefore, to understand if mechanically linking the active use of the arms with the legs disrupted walking balance, we carried out a secondary analysis by quantifying the variability in step width, step length, and step time.”**

**However, as recommended, we revised the Discussion section to include that our small sample size is a limitation of this study:**

**Lines 206-207: “These comparisons should be taken with caution given our small sample size and the limited data that is reported for stepping variability in the clinical and gait rehabilitation literature.”**

**Comment 2:** The finding reported in this study is that the rope pulley system marginally increased step time variability but not all other parameters of interest. I think this is comparably weak findings with a small sample size. The variability parameters are all linear variability and non-linear gait dynamics will be a great addition to the current weak findings.

**Response: We understand your suspicion of the findings in respect to the statistical significance and small sample size. To alleviate such concerns (which we also had), we use effect sizes. Comparing our effect sizes to that in the literature helps with making meaningful interpretations since it tells us the magnitude or strength of the findings (i.e., an increase in variability). Additionally, effect sizes are independent of sample size and provides meaningful comparisons of various studies to one another despite differing sample sizes.**

**To make these points clearer, we have added the following sentence (underlined text) to help readers understand why the use of comparing effect sizes is a reasonable gauge given our small sample size:**

**Lines 166-169: “To assess whether the statistical increase in step time variability reflected a meaningful change, we used its effect size to interpret the relative magnitude to which walking balance was disrupted. Additionally, the value of calculating effect sizes is that they are independent of sample size and allows us to compare findings from various studies.”**

**We appreciate the recommendation of including non-linear gait dynamics, however, we feel that such analysis is far beyond the scope of the original motivation of our paper. In our Introduction (see lines 57-74), we motivated the use of linear variability based on clinical literature that provides compelling evidence that linear variability measures are simple metrics that have shown to be good clinical correlates for walking balance (Day et al. 201). Additionally, in a comprehensive review of assessing stability of human locomotion, Bruijn et al. (2013) finds that stepping variability shows good validity for fall probability. We believe the literature supports that stepping variability is a useful metric to understand balance. Furthermore, linear variability metrics are often more easily understood by clinicians compared to non-linear variability metrics.**

**Comment 3:** Please provide the rationale for the hypothesis. How can an increase in either one of three parameters of variability be enough to indicate disruption in walking balance?

**Response: Thank you for inquiring about the rationale.**

**Since our method of mechanically coupling the arms and legs during treadmill walking is novel, there is no existing literature with the same device or mechanical strategy that would help inform a strong rationale for the increase in all three stepping variability parameters. To provide a rationale for a possible balance disruption, we provided a reference by Siragy et al (2020) where the intentional and voluntary arm swing kinematics is similar to the strategy adopted by our subjects with the rope-pulley device (Vega and Arellano, 2021). In the study by Siragy et al (2020), they found an increase in the variability of step width, length, and time. Therefore, we suspected a possible disruption to walking balance when using our device. However, arm swing strategy is just one part of what occurred in our study (Vega and Arellano, 2021). The physical connection between the arms and legs led to both a decrease in propulsive forces and an increase in braking forces by the legs during walking. Given the complexity of these multifactorial outcomes, it was impossible to understand which factors led to what kind of balance disruption (e.g. medial-lateral, posterior-anterior, temporal). Therefore, we could not provide a strong rationale for an increase in all three variability stepping parameters. However, we suspected that if balance was disrupted, then at least one of these parameters would increase and our findings from exploring possible disruptions would help inform future studies.**

**Furthermore, when it comes to stepping variability during human walking, the literature shows that one variable may be more sensitive than others when distinguishing fallers vs non-fallers (see review paper by Hamacher et al 2011). In visual perturbation studies, they have shown an increase in both stepping variables, but one seems to be more sensitive than the other (Collins and Kuo 2013; Bauby and Kuo, 2000). For example, Collins and Kuo (2013) have shown that when subjects walk with their eyes closed (a visual perturbation), they exhibit an increase in both step width and length variability when compared to walking with their eyes open. Yet, after accounting for speed, they found step width variability to be four times greater than step length variability. They interpret these findings to suggest that step variability may have independent components separable in both direction and time. This motivated us to examine walking balance based on stepping variability in the temporal and spatial domains. We used step time variability to quantify the temporal aspect and used variability of step length and step width to quantify the spatial aspect. Furthermore, since we had no prior knowledge of balance when using this arm-leg rope-pulley device but suspected a possible disruption, we explored all three stepping variability parameters and hypothesized that an increase in variability in at least one of these stepping parameters would indicate disruption to walking balance. The magnitude of disruption is later discussed using effect sizes which we ultimately conclude is minimal to moderate disruption.**

**As recommended, we have revised the introduction to provide a clearer rationale for the hypotheses by adding the following two sentences (underlined text):**

**Lines 75-86: “Understanding if, and to what extent, balance was compromised will help determine if linking the arms and legs during treadmill walking could feasibly translate to clinical users undergoing gait rehabilitation. Helping patients maintain balance while practicing their stepping motion is critical for walking recovery (19,20), reducing fear of falling (21,22), and preventing falls (23). Ideally, our arm-leg rope pulley system would not disrupt walking balance, but our observations of abnormal arm swinging suggest otherwise. Therefore, we wanted to understand if physically coupling of the arms and legs during walking may have influenced balance. Given the novelty of this device, we did not have any a priori knowledge as to which stepping parameter(s) would exhibit an increase in variability. For this reason, we hypothesized that walking with our arm-leg rope pulley system would increase the variability in either step width, step length, and/or step time. If walking with our arm-leg rope pulley system did disrupt balance, we expect an increase in the variability of at least one of these stepping parameters.”**

**References*:**

**Siragy T, Mezher C, Hill A, Nantel J. Active arm swing and asymmetric walking leads to increased variability in trunk kinematics in young adults. Journal of Biomechanics. 2020 Jan;99.** [**https://doi.org/10.1016/j.jbiomech.2019.109529**](https://doi.org/10.1016/j.jbiomech.2019.109529)

**Hamacher D, Singh NB, van Dieën JH, Heller MO, Taylor WR. Kinematic measures for assessing gait stability in elderly individuals: a systematic review. Journal of The Royal Society Interface. 2011 Dec 7;8(65).** [**https://doi.org/10.1098/rsif.2011.0416**](https://doi.org/10.1098/rsif.2011.0416)

**Bauby CE, Kuo AD. Active control of lateral balance in human walking. Journal of Biomechanics. 2000 Nov;33(11).** [**https://doi.org/10.1371/journal.pone.0073597**](https://doi.org/10.1371/journal.pone.0073597)

**Collins SH, Kuo AD. Two Independent Contributions to Step Variability during Over-Ground Human Walking. PLoS ONE. 2013 Aug 28;8(8).** [**https://doi.org/10.1371/journal.pone.0073597**](https://doi.org/10.1371/journal.pone.0073597)

***Please note that all of these references have been mentioned in the manuscript.**

**Comment 4:** I understand that it would be not feasible to collect more data as this is a secondary data analysis. Anyhow, please report the power of current analysis which may provide more clear view of your acknowledgement of the small sample size as the limitation of this study.

**Response: We appreciate your understanding that this paper is based on secondary data analysis. If we understand your suggestion correctly, the recommendation is to perform a post hoc power analysis. To address your suggestion, we consulted with our “in-house” Biostatistician, Dr. Daniel P. O’Connor (**[**https://grants.hhp.uh.edu/doconnor/**](https://grants.hhp.uh.edu/doconnor/)**) and we were advised that conducting a statistical power analysis based on the data that we have already collected and analyzed is conceptually flawed. We were pointed to key references on this topic (please see list below).**

**References:**

**Cohen J. Statistical power analysis for the behavioral sciences. 2nd ed. Mahwah, NJ: LEA, 1988**

**Zhang, Y. et al. Post hoc power analysis: is it an informative and meaningful analysis? Gen Psychiatry 32, e100069 (2019).** [**http://dx.doi.org/10.1136/gpsych-2019-100069**](http://dx.doi.org/10.1136/gpsych-2019-100069)

**Significance testing (as we have done by testing the mean difference in average and variability metrics between walking without the device and with the device) is not designed to determine the statistical power of the test. As explained in these key references, we have learned that the appropriate way to carry out a statistical power analysis is during the design phase of a prospective study. While many notable statisticians have made a case that “post hoc” power analyses do not make conceptual sense, Zhang et al. (2019) also addressed the question “if such power estimates [post hoc] can inform power for [failing to detect] significant results”. The power equation (please see equation 3 in Zhang et al., 2019) is based on the “population” mean difference and standard deviations, not the random sample that we have collected. Thus, using parameters from a random sample in the power equation for parameters from the population is conceptually flawed. Furthermore, the authors note that “analytically, [the population mean] and [population standard deviation] are population-level constants, whereas the observed means and standard deviations are random quantities and vary from sample to sample”.**

**Instead of conducting a post hoc power analysis, Dr. O’Connor (biostatistician consultant) suggested that we perform two sensitivity analyses using GPower. The first sensitivity analyses requires the input parameters of sample size, power and statistical test to output the detectable effect size under these two conditions. This type of analysis helps us determine the effect size that would be detectable in our sample size of 8 and with 80% power. Please note that we do not know a priori the true effect size and this type of analysis (where power value is an input parameter and therefore conceptually in line with power analyses) allows for interpreting the results in the context of sample size and the magnitude of the effect size we can detect with our experimental design.**

**Figure 1.** Sensitivity analysis for determining the effect size that would be detectable across various sample sizes with 80% power, α = 0.05, and a one-tailed dependent t-test. In our study of n = 8, the detectable effect size for our parametric variables step width and step time variability would yield a detectable effect size of at least 0.98 or above.

**Figure 2.** Sensitivity analysis for determining the effect size that would be detectable across various sample sizes with 80% power, α = 0.05, and a one-tailed Wilcoxon signed-rank test (matched pairs). In our study of n = 8, the detectable effect size for our non-parametric variable step length variability would yield a detectable effect size of at least 1.00 or above.

**Based on the sensitivity analysis with 80% power and using a one-tailed dependent t-test (Figure 1), we can detect an effect size of 0.98 and above with a sample size of *n* = 8. Therefore, we can conclude that our effect size for step time variability (effect size = 1.286) falls within this range of 0.98 and above, and therefore, our study has sufficient power to detect this effect size. This suggests that the temporal component of balance is most disrupted with the use of this simple device. For step width variability, our effect size is 0.146 and does not fall within the calculated range. Therefore, our study did not have sufficient power to detect an effect size of 0.146 for step width variability. To be able to detect an effect size of ~0.14 with 80% power, we would need at least 293 subjects (Figure 1). For step length variability, we conducted another sensitivity analysis with 80% power and using a one-tailed Wilcoxon signed rank test (Figure 2) since this variable did not meet the assumption of normality. Based on the sensitivity analysis, we can detect an effect size of 1.0 and above with a sample size of *n* = 8. Since our effect size for step length variability is 0.385 and does not fall within the calculated range of 1.0 and above, we conclude that our study did not have sufficient power to detect an effect size of 0.385 for step length variability. To be able to detect an effect size of ~0.38 with 80% power, we would need at least 46 subjects (Figure 2).**

**Since the effect size of step width and step length variability do not fall within the calculated range of detectable effect sizes based on the sensitivity analysis, this means that if there were actually a *true* increase in variability in these two metrics, then our sample size of 8 would not have captured this. Therefore, our findings for step width and length *may not* be adequately powered and would require a larger sample size.**

**With the first sensitivity analyses, we were able to determine if our variability variables are sufficiently powered with a sample size of 8. In a second sensitivity analysis, we can use “meaningful” effect sizes to determine the appropriate sample size needed for a future study to detect a minimal effect size considered meaningful based on data from the literature (see figure 3 below). Here, the “meaningful” effect sizes we chose are the ones based by Callisaya et al. (2011) whose findings suggest that differences in step time variability and step length variability are most notable in distinguishing fallers and non-fallers. Therefore, we use these data as a preliminary gauge to interpret our findings. We also chose Madehkhaksar et al. (2018) whose findings suggest that step width variability is affected during a forward treadmill perturbation and use this as a preliminary gauge to interpret our findings as well. These two studies have been discussed in the manuscript to compare our effect sizes for meaningful interpretations and now, we will use their effect sizes to help determine the appropriate sample size needed for a future study. The data and calculated effect sizes are reported in S1 file. We also list the effect size for each variable below.**

**Figure 3.** Sensitivity analysis for determining the sample size needed across various effect sizes with 80% power, α = 0.05, and a one-tailed dependent t-test. Based on the effect sizes gathered from the literature for step time, length and width variability, the analysis suggests a study would require between 10 and 49 subjects to be able detect these effect sizes with sufficient power.

***“Meaningful” effect sizes based on previous literature:***

i. ***Callisaya et al. (2011)***

***Step time variability effect size is 0.486. Sensitivity analysis suggests that we need 28 subjects.***

***Step length variability effect size is 0.369. Sensitivity analysis suggests that we need 49 subjects.***

ii. ***Madehkhaksar et al. (2018)***

***Step width variability effect size is 0.845. Sensitivity analysis suggests that we need 10 subjects.***

**Based on the effect sizes from these studies and the sensitivity analysis (Figure 3), a study with 80% power would require between 10 and 49 subjects to be able to detect “meaningful” effect sizes (0.369-0.845). However, a sample size of 49 is atypical of current sample sizes observed in the gait literature, specifically in those with spinal cord injury, but a sample size of 10 may be reasonable.**

**We hope that our sensitivity analyses (included as supplementary material, see S2 file) and additional interpretation satisfies the reviewer’s concerns. We have revised the Discussion section of our manuscript with the following:**

**Lines 216-247: “To further address the limitation of our small sample size, we carried out sensitivity analyses in GPower (please see S2 File for more details) to determine if the findings from our variability variables were adequately powered. Our analysis parameters were set to 80% power, α = 0.05 and a one-tailed dependent t-test or a one-tailed Wilcoxon signed rank test. Based on the analyses, a sample size of 8 indicates that the minimum effect size that can be detected are 0.978 for step width variability and 1.007 for step length variability. The effect sizes found in our study (step width variability ES = 0.146 and step length variability ES = 0.385) fall short of the minimum effect size that can be detected with 80% power. Therefore, we caution that our findings for step width and length variability may not be adequately powered with our sample size of 8. However, the sensitivity analysis reveals that our findings for step time variability appears to be adequately powered since our effect size (1.286) is above the minimum effect size of 0.978 detectable at 80% power. We interpret this to suggest that the temporal component of walking balance is most disrupted with the use of the arm-leg rope-pulley system. Yet, it is possible that the spatial component may have been disrupted but was not detectable with our sample size of 8 subjects.**

**To determine the appropriate sample size needed for a future study, we carried out another sensitivity analysis (S2 File). For this analysis, we used “meaningful” effect sizes based on data gathered from the literature to determine the sample size needed for a study with sufficient power (parameters were set to 80% power, α = 0.05, and a one-tailed dependent t-test). The meaningful effect size for each stepping variable were gathered from two studies that investigated stepping variability in fallers versus non-fallers (18) and under treadmill perturbations (26). Given the limited data, these studies were chosen as a reasonable gauge because their findings suggest that 1) increases in step time and step length variability are most notable in distinguishing fallers from non-fallers (18) and 2) step width variability increased under a forward treadmill perturbation (26), which is analogous to the effects of our arm-pulley device. This sensitivity analysis reveals that the sample size for a sufficiently powered study would require between 10 to 49 subjects. We suspect that for individuals undergoing gait rehabilitation (e.g., patients with a spinal cord injury), such effect sizes would be much greater in magnitude, and therefore, this may be a conservative estimate of sample size. A sample size of 49 is atypical in experiments involving individuals with an incomplete spinal cord injury, but a sample size of 10 may be too small. Considering the resources, time, and challenges with subject recruitment of this clinical population (27), we feel that a sample size of n = 20 would be reasonable for a future study investigating walking balance using linear variability measures.”**

**Reference added:**

**(27) Blight AR, Hsieh J, Curt A, Fawcett JW, Guest JD, Kleitman N, et al. The challenge of recruitment for neurotherapeutic clinical trials in spinal cord injury. Spinal Cord. 2019 May 8;57(5).**

**Comment 5:** How were the variability parameters computed? Provide citation for the way of normalized standard deviation with leg length. Standard deviation also needs to be normalized with average value, which is a common computation of gait variability called coefficient of variation. Please consider using this parameter and justify the current data processing method.

**Response: Thank you for the suggestion. We recognize that both coefficient of variation and standard deviation are common methods to compute step variability (Hamacher et al. 2011). Variability measures were computed using the standard deviation of all 288 steps for each stepping parameter (i.e., step width, length and time). For both average and standard deviation values, step length and width were normalized to leg length to account for body size, i.e., we used a scaling approach. Step time values were normalized by the square root of leg length divided by gravity, another scaling approach for the temporal component. These scaling approaches are recommended by AL Hof (1996) and we have referenced the paper in the methods section of our manuscript. Additionally, such data processing methods have been previously performed in studies. For example, Bauby and Kuo (2000) computed variability using standard deviation across steps for lateral and fore-aft directions and then normalized these values by leg length. We do not normalize the standard deviation about the average rather, we normalize our variables to body size because there is a biomechanical basis that underlies a physical law that determines how animals with different sizes can move about in the world (Biewener, A and Patek, S. Animal Locomotion. 2nd ed., 2018, R. McNeill Alexander (https://press.princeton.edu/books/paperback/9780691126340/principles-of-animal-locomotion, and John Tyler Bonner (https://press.princeton.edu/books/paperback/9780691152332/why-size-matters).**

**While some studies do report both standard deviation and coefficient of variation alongside each other, we are not aware of a study that shows one is more advantageous than the other. Given that our approach takes into account differences in body size and that we report effect sizes, we do not feel it is necessary to incorporate coefficient of variation into our analysis.**

**As recommended, we revised the text to address our normalization approach by adding the references mentioned:**

**Lines 114-115: “For each subject, we normalized step width, step length, and their variability by dividing each metric by leg length (LL) and expressed these values as a percentage (24, 8).”**

**References:**

**(24) Hof AL. Scaling gait data to body size. Gait & Posture. 1996 May; 4(3).**

**(8) Bauby CE, Kuo AD. Active control of lateral balance in human walking. Journal of Biomechanics. 2000 Nov;33(11).**

**Comment 6:** Please provide the interpretation of Figure 2. What do you mean by a representative subject?

**Response: By representative subject, we mean the data from one subject is being shown. We report individual time series data during normal walking and when walking with the arm-leg rope pulley system to help visualize the data trends of all three stepping parameters for each subject (step length, width and time).**

**We understand that “representative” may be misleading since subjects responded differently. Thank you for pointing this out. We have revised the text and figure caption with the following to avoid any misinterpretation:**

**Lines 117-118: “To help visualize the time series data for each stepping parameter across 288 steps, Figure 2 illustrates the time series data for one subject.”**

**Lines 146-147: “Fig 2. Time series data (*n* = 1). Solid circles represent values for each step over a series of 288 consecutive steps for one subject.”**

**Comment 7:** Standard deviation-based variability parameters are linear variability of human gait.  Please consider additional analysis of non-linear analysis of gait dynamics to strengthen the current findings. Non-linear gait analysis is appropriate for this type of time-series gait data. While the linear variability of gait represents the magnitude of the step-to-step fluctuation, dynamics of gait changes (alterations in the fractal pattern) is a great addition to the study of gait variability (Hausdorff, 2005, DOI: [https://doi.org/10.1186/1743-0003-2-19](https://urldefense.com/v3/__https:/doi.org/10.1186/1743-0003-2-19__;!!LkSTlj0I!DyCIhVH_ekEeO1CsZ_dnQXePJlqBfCWBuImfGEGidAeFvViOVv-62X1cQO7W4n_LLt4Pa2ZBgRJvNOU$)). Although fractal analysis of gait variability used to conducted with time series gait data with > 600 strides, Kuznetsov and Rhea (2017) demonstrated that it is possible with relatively short gait time series (100 - 200 strides) (DOI: [10.1371/journal.pone.0174144](https://urldefense.com/v3/__https:/dx.doi.org/10.1371*2Fjournal.pone.0174144__;JQ!!LkSTlj0I!DyCIhVH_ekEeO1CsZ_dnQXePJlqBfCWBuImfGEGidAeFvViOVv-62X1cQO7W4n_LLt4Pa2ZB8vkXEss$)). If this additional analysis of gait variability is not conducted, please provide justification that three variables of interest are adequate indicators of gait instability. Also discuss and acknowledge that the current analysis of gait variability is limited to linear analysis and that fractal analysis of gait variability was not considered due to some limitation but will strengthen the whole picture of gait variability altered by the rope pulley system in a future study.

**Response: Thank your recommendation and thoughtful guidance on non-linear gait dynamics. We recognize that there are various methods to analyze balance from simple metrics such as stepping variability to more complex measures such as Lyapunov exponent and fractal analysis (we were guided by the comprehensive review published by Bruijn et al. (2013) who compare these different measures). While there is value in various non-linear gait analyses, we feel that carrying out this additional analysis is out of the scope of our original motivation and would require an entirely different paper than intended. We addressed the use of linear gait variability instead of non-linear gait variability in response to Comment 2. As a field, we agree that it will be important to understand which metrics are most useful in capturing balance disruptions and to have a fundamental understanding of the magnitude of effect size that is functionally meaningful.**

**Based on the reviewer’s comment, we revised the text to indicate that the focus on linear gait variability is a limitation of this study and that adding non-linear gait variability would insightful.**

**Lines 210-213: “It should also be noted that the analyses of this study were focused on linear analyses of gait variability. It may be helpful to consider other metrics (e.g., fractal analysis of gait variability, Lyapunov exponent, etc.) that are based on non-linear analyses in future studies, which may capture potential disruptions to balance in ways that linear analyses do not.”**
